# Prevalence and Molecular Characteristics of Avian Pathogenic Escherichia coli in “No Antibiotics Ever” Broiler Farms

**DOI:** 10.1128/Spectrum.00834-21

**Published:** 2021-12-08

**Authors:** Courtney A. Fancher, Hudson T. Thames, Mary Gates Colvin, Mercedes Smith, Alyssa Easterling, Nikhil Nuthalapati, Li Zhang, Aaron Kiess, Thu T. N. Dinh, Anuraj Theradiyil Sukumaran

**Affiliations:** a Department of Poultry Science, Mississippi State University, Starkville, Mississippi, USA; b Department of Animal and Dairy Sciences, Mississippi State University, Starkville, Mississippi, USA; c Virginia-Maryland College of Veterinary Medicine, Blacksburg, Virginia, USA; d University of Missouri College of Veterinary Medicine, Columbia, Missouri, USA; e Prestage Department of Poultry Science, North Carolina State University, Raleigh, North Carolina, USA; The National University of Singapore and the Genome Institute of Singapore

**Keywords:** APEC, broiler, virulence, antimicrobial susceptibility, colibacillosis, *Escherichia coli*, NAE, antibiotic resistance, serogroup

## Abstract

Avian pathogenic Escherichia coli (APEC) causes significant economic and welfare concerns to the broiler industry. For several decades, prophylactic supplementation of antimicrobial growth promoters was the primary method to control APEC; however, the recent shift to no antibiotics ever (NAE) production has increased colibacillosis incidence. The objectives of this study were to determine the influence of season, flock age, and sample type on the prevalence and virulence of E. coli and to identify the serogroups and antimicrobial susceptibility of virulent and nonvirulent E. coli in NAE broiler farms. Litter, feces, cloacal swabs, and tracheal swabs were collected from 4 NAE farms during spring and summer seasons, and E. coli was isolated and confirmed by PCR. Confirmed E. coli isolates were tested for 5 APEC-virulence-associated genes (VAGs) using quantitative PCR (qPCR). Further, E. coli isolates with all five VAGs (100 isolates) and E. coli isolates without any VAGs (87 isolates) were screened against 11 antimicrobials through Kirby-Bauer disk diffusion assay, and their serogroups were tested using PCR. Data were analyzed using the GLIMMIX procedure of SAS 9.4, and statistical significance was determined at a *P* value of *≤*0.05. Overall, the prevalence of E. coli was not affected by season, flock age, or sample type. However, the prevalence of all tested VAGs decreased from spring to summer (*P ≤ *0.002). The frequency of resistance was highest for tetracycline, and serogroups O8 (31%) and O78 (11%) were most frequent in virulent E. coli. In conclusion, there is a high prevalence of virulent E. coli in NAE farms, especially in the spring season.

**IMPORTANCE** Avian pathogenic Escherichia coli causes one of the most detrimental bacterial diseases to the United States poultry industry, colibacillosis. Colibacillosis leads to decreased performance, early mortality, and subsequent production loss. Previously, colibacillosis was largely mitigated by the use of antimicrobial growth promoters. Due to concerns about antimicrobial resistance, the use of these promoters has been largely removed from the broiler industry. With recent shifts in the poultry industry to NAE broiler production, there is an increase in bacterial disease and mortality. We do not know how this shift to NAE affects APEC prevalence within broiler farms. Therefore, in the current study, we attempted to assess the prevalence and virulence of E. coli within an antibiotic-free broiler environment, assessed antimicrobial susceptibility, and identified the serogroups of virulent and nonvirulent E. coli.

## INTRODUCTION

Escherichia coli is a Gram-negative bacterium of the *Enterobacteriaceae* family present in many environments, including the gastrointestinal tract (GIT) and mucosal surfaces of poultry. E. coli strains can be found readily in the surrounding broiler environment and are also released into the environment via feces (1, 2). Extraintestinal pathogenic E. coli strains (ExPEC) are E. coli strains that cause disease outside the GIT (3). Avian pathogenic E. coli (APEC) is a subset of ExPEC that causes localized and systemic infections in poultry that result in significant morbidity and mortality and subsequent production loss (4). Disease caused by APEC in broilers is collectively referred to as avian colibacillosis and is the most common infectious bacterial disease in poultry, often characterized by lesions within the air sacs, the heart, and the liver, followed by septicemia and death (1, 5, 6). Colibacillosis causes decreased performance, early morbidity, and mortality that increase production losses (5).

Most APEC isolates contain plasmid-linked virulence genes acquired through horizontal gene transfer (1, 7). No single, distinct virulence factor distinguishes APEC from other E. coli strains (8). However, Johnson and others identified specific plasmid-carried virulence-associated genes (VAGs) *hlyF*, *ompT*, *iroN*, *iss*, and *iutA* that are frequently found in APEC strains and suggested that these genes could be used to differentiate APEC from nonpathogenic E. coli (1). Moreover, APEC isolates belong mostly to serogroups O1, O2, O8, O15, O18, O35, O36, O78, O88, O109, O111, and O115, among the 188 O groups identified for E. coli, with O1, O2, and O78 most correlated with colibacillosis (9–11). However, designation to a particular serogroup does not always reflect the virulence of the isolate in question (12). Therefore, serogrouping used in conjunction with other diagnostic tools, such as virulence gene testing, is needed to better detect APEC.

The use of antimicrobial growth promoters (AGPs) was the main barrier against the incidence of colibacillosis. They protect birds by modifying gut microbiota, reducing GIT inflammation, and improving the physical health of the GIT (13). However, due to increased concerns over antimicrobial resistance, broiler operations have limited their antibiotic usage. In 2011, the annual Agricultural Resource Management Survey concluded that 48% of broiler grow-out operations raised broilers without antibiotics and provided antibiotics only when birds were sick (14). Recently, it was estimated that over 50% of the broiler industry raises broilers without any antibiotics (https://www.nationalchickencouncil.org/questions-answers-antibiotics-chicken-production-2/#:~:text=As%20of%;20April%202019%2C%20more%20than%2050%25%20of,those%20questions%20and%20address%20some%20of%20those%20concerns). Broilers raised under NAE policy are not allowed to receive any antimicrobials in feed, water, supplementation, or injection at any point in the bird’s lifetime (15). Removal of AGPs has increased mortality within NAE broiler production by 25% to 50% compared to that in conventional production (16). The mortality rate in NAE is 4.2%; whereas conventional broiler production has a mortality rate of only 2.9% (13). With greater disease incidence and mortality rate, it is crucial to understand the prevalence and virulence characteristics of E. coli, one of the most common avian pathogens, within NAE farms.

Information on APEC prevalence or virulence in commercial NAE broiler production is lacking. Therefore, the objectives of this study were to (i) determine the prevalence, virulence, and antimicrobial susceptibility of E. coli in NAE commercial broiler farms and (ii) determine the influence of season, age of flock, and type of sample on the prevalence and virulence of E. coli in NAE commercial broiler farms.

## RESULTS

### E. coli prevalence and virulence.

In total, 512 samples, including 128 litter samples, 128 fecal samples, 128 cloacal swabs, and 128 tracheal swabs, were collected from the four NAE farms. A total of 2,432 presumptive E. coli isolates were collected from the MacConkey agar plates. Of the samples collected in the current study, 93% (477/512) tested positive for E. coli. Of the 477 E. coli*-*positive samples, 76.7% (366/477) were positive for *iroN*, 72.3% for *ompT* (345/477), 94.3% for *hlyF* (450/477), 82.4% for *iss* (393/477), and 90.8% samples were positive for *iutA* (433/477). Moreover, 68.1% (325/477) samples were positive for all five VAGs, and only 12.6% (60/477) samples were negative for all five VAGs. A total of 2,127 isolates were confirmed as E. coli with the presence of *ybbW*. Of the 2,127 E. coli isolates, 29.7% (631/2,127) of isolates tested positive for all five VAGs and only 4.1% (87/2,127) isolates tested negative for all five of the VAGs.

**FIG 1:**
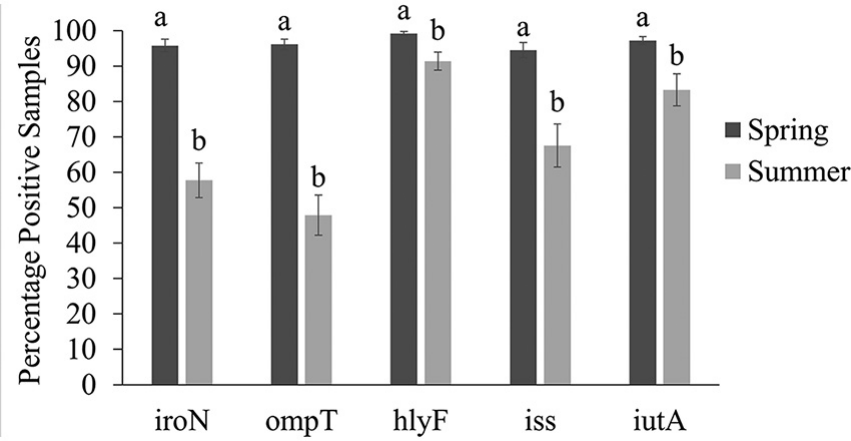
Prevalence of E. coli with virulence genes *iroN*, *ompT*, *hlyF*, *iss*, or *iutA* in samples collected from NAE broiler farms during spring and summer seasons. The prevalence is expressed as percentage of samples positive for E. coli with each gene (mean ± standard error of the mean [SEM]). Within each group, means without common letters differ (*P ≤ *0.05).

**FIG 2:**
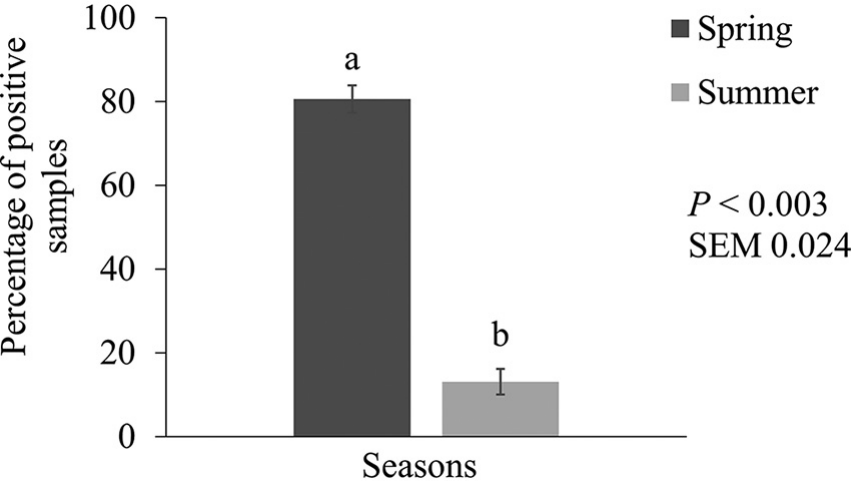
Prevalence of E. coli (mean ± SEM) containing all five virulence-associated genes, *iroN*, *ompT*, *hlyF*, *iss*, *and iutA*, in NAE broiler farms during spring and summer seasons. The prevalence is expressed as percentage of samples positive among all type of samples collected, including litter, feces, and cloacal and tracheal swabs. Means without common letters differ (*P ≤ *0.05).

Overall, for all tested VAGs, there was a greater prevalence of E. coli with VAGs in samples collected in the spring growing season than in those collected in the summer growing season (Fig. 1; *P ≤ *0.002). The prevalence of *iroN* in E. coli-positive samples decreased from spring (95.8%) to summer (57.7%, *P < *0.001). The prevalence of *ompT* in E. coli-positive samples also decreased from spring (96.1%) to summer (47.9%, *P < *0.001). The prevalence of *hlyF* in E. coli-positive samples decreased slightly from spring (99.2%) to summer (91.4%; *P < *0.002). The prevalence of *iss* and *iutA* genes in E. coli-positive samples also decreased from spring (94.5% and 97.2%) to summer (67.6% and 83.3%, respectively; *P < *0.001). Finally, 80.6% of the samples collected in the spring contained E. coli with all five VAGs compared to only 13.0% of the samples collected in the summer (*P < *0.003; Fig. 2).

**FIG 3:**
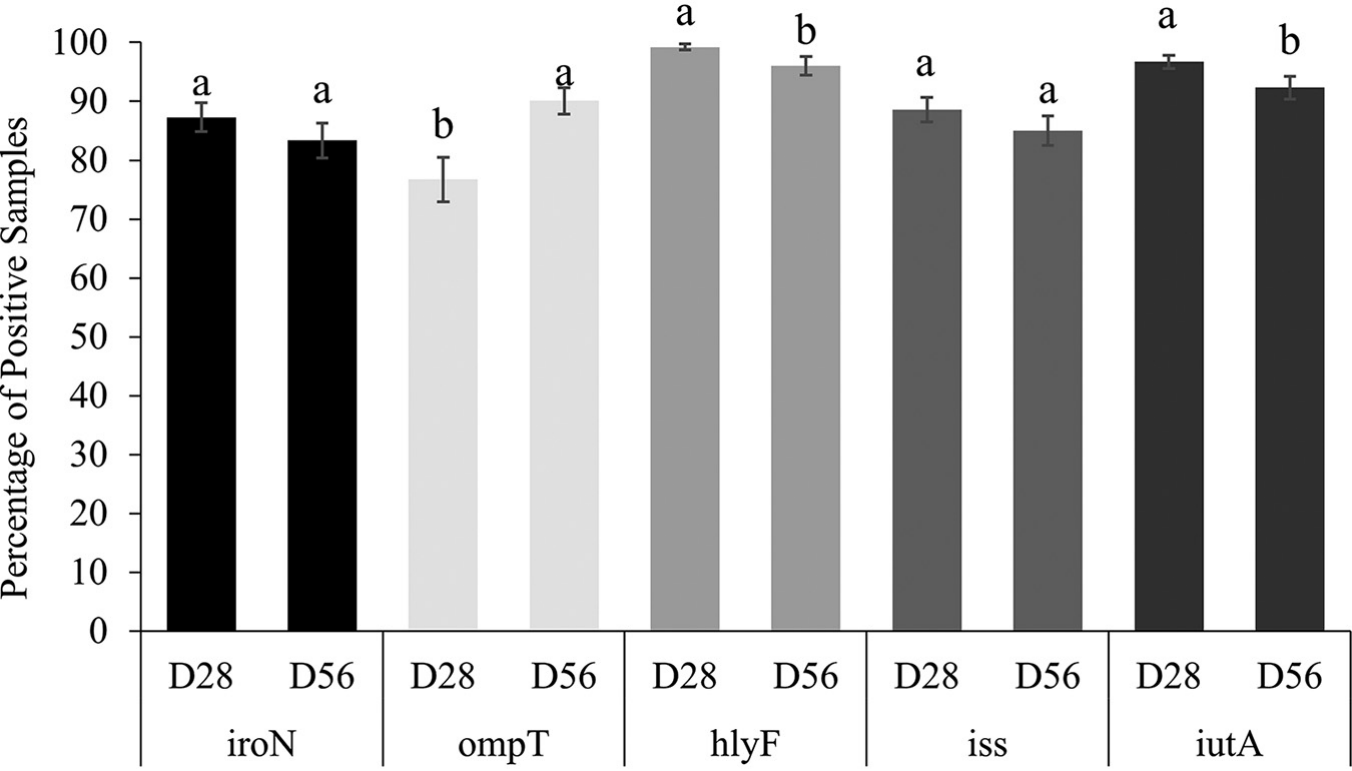
Prevalence of virulence genes (mean ± SEM), *iroN*, *ompT*, *hlyF*, *iss*, *and iutA*, on day 28 and day 56 of flock cycle, averaged across seasons and sample types. For each gene, means without common letters differ (*P ≤ *0.05).

The flock age influenced the prevalence of *ompT*, *hlyF*, and *iutA* (Fig. 3). The prevalence of *ompT* in E. coli-positive samples increased from 76.7% on day 28 to 90.1% on day 56 (*P < *0.001). Virulence gene *hlyF* had the greatest prevalence in E. coli-positive samples with 99.2% on day 28; however, prevalence decreased to 96.0% on day 56 (*P < *0.002). The prevalence of *iutA* in E. coli-positive samples decreased from 96.7% on day 28 to 92.3% on day 56 (*P < *0.021). Prevalence of *iroN* and *iss* genes was not influenced by the day of flock. The prevalence of *iroN* was 87.3% on day 28 and 83.3% on day 56 (*P = *0.236). The prevalence of *iss* was 88.6% on day 28 and 85.0% on day 56 (*P = *0.238).

**FIG 4:**
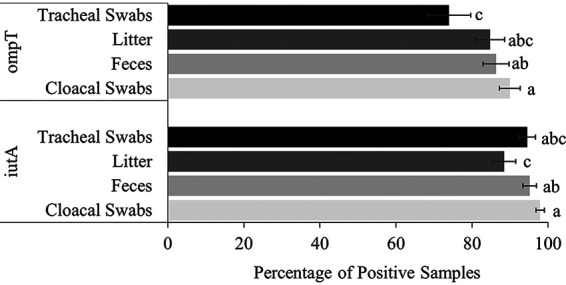
Prevalence of virulence genes (mean ± SEM) *ompT* and *iutA* in litter, feces, cloacal swabs, and tracheal swabs collected from NAE farms, averaged across seasons and age of flock. For each gene, means without common letters differ (*P ≤ *0.05).

Sample type influenced only VAGs *ompT* and *iutA* (Fig. 4). The prevalence of *ompT* was greatest in cloacal swabs at 90.0%, with less prevalence in feces, litter, and tracheal swabs at 86.3%, 84.8%, 73.9%, respectively (*P ≤ *0.045). For *iutA*, the prevalence in E. coli-positive samples was greatest in the cloacal swabs at 97.9%, with less prevalence in the feces, tracheal swabs, and litter at 95.2%, 94.5%, and 88.5%, respectively (*P ≤ *0.041).

### E. coli antimicrobial susceptibility.

**FIG 5:**
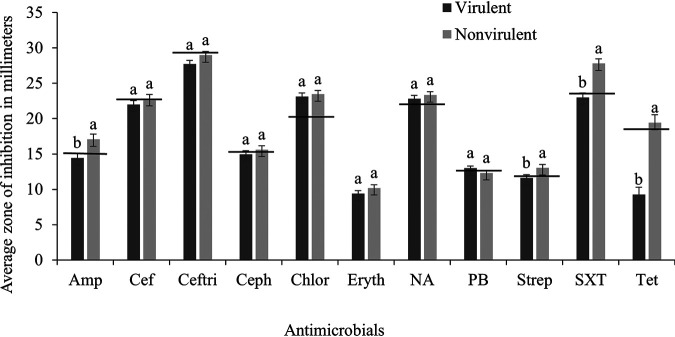
Average zone of inhibition (mean ± SEM) of virulent (isolates with all 5 virulence genes; 100 isolates) and nonvirulent (isolates without any of the tested virulence genes; 87 isolates) E. coli against 11 classes of antimicrobials. Horizontal lines indicate the lower limit of the susceptibility range established by Clinical & Laboratory Standards Institute (CLSI) for E. coli ATCC 25922. The average zones of inhibition below the set line are classified as resistant, and those above are considered susceptible. For each antimicrobial, means without common letters differ (*P ≤ *0.05). AMP, ampicillin; CEF, cefoxitin; CEFTRI, ceftriaxone; CEPH, cephalothin; CHLOR, chloramphenicol; ERYTH, erythromycin; NA, nalidixic acid; PB, polymyxin B; STREP, streptomycin; SXT, sulfamethoxazole/trimethoprim; TET, tetracycline.

The CLSI-established susceptibility ranges are reported in Table 1. Virulent E. coli overall had a greater frequency of antimicrobial resistance, except to cefoxitin, compared to nonvirulent E. coli (Table 2). For resistance to tetracycline, virulent E. coli exhibited resistance with a frequency of 62% (62/100 isolates) and a zone of inhibition (ZI) of 9.26 mm, more than that of nonvirulent E. coli at 18.4% (16/87 isolates) and 19.4 mm (*P < *0.001). For resistance to sulfamethoxazole/trimethoprim, virulent E. coli exhibited antimicrobial resistance with a frequency of 29% (29/100) and a ZI of 23.0 mm, compared to that of nonvirulent E. coli at a frequency of 8.05% (7/87) and ZI of 27.29 mm (*P ≤ *0.001). For resistance to streptomycin, virulent E. coli had resistance frequency at 39% and a mean ZI of 11.6 mm and classified as resistant, compared to nonvirulent E. coli at a frequency of resistance at 25.3% (22/87) and a ZI of 13.0 mm (*P ≤ *0.046). For antimicrobial resistance to ampicillin, virulent E. coli exhibited a frequency of 30% (30/100) and ZI of 14.4 mm, compared to the nonvirulent E. coli that had a frequency of 17.2% (15/87) and a ZI of 17.1 mm (*P ≤ *0.042). As in Fig. 5, virulent isolates were resistant to 7 of the 11 antimicrobials tested and nonvirulent E. coli isolates were resistant to only 2 out of 11 antimicrobials tested.

**TABLE 1:** Primer sequences and gene descriptions for five virulence-associated genes of E. coli: *iroN*, *ompT*, *hlyF*, *iss*, and *iutA* (1)

Gene	Amplicon size (bp)	Sequence (5′-3′)	Description
*iroN*	553	AATCCGGCAAAGAGACGAACCGCCTGTTCGGGCAACCCCTGCTTTGACTTT	Salmochelin siderophore receptor gene
*ompT*	496	TCATCCCGGAAGCCTCCCTCACTACTATTAGCGTTTGCTGCACTGGCTTCTGATAC	Episomal outer membrane protease gene
*hlyF*	450	GGCCACAGTCGTTTAGGGTGCTTACCGGCGGTTTAGGCATTCCGATACTCAG	Putative avian hemolysin
*iss*	323	CAGCAACCCGAACCACTTGATGAGCATTGCCAGAGCGGCAGAA	Episomal increased serum survival gene
*iutA*	302	GGCTGGACATCATGGGAACTGGCGTCGGGAACGGGTAGAATCG	Aerobactin siderophore receptor gene

**TABLE 2:** Target genes and primers used for identification of E. coli O-serogroups using PCR

O-genotype	Associated O serogroup	Target gene	Primer name	Sequence (5′–3′)	Size (bp)	Reference
Og1	O1	*wzx*	Og1-F	GTGAGCAAAAGTGAAATAAGGAACG	1098	43
			Og1-R	CGCTGATACGAATACCATCCTAC		
Og2	O2	*wzx*	Og2-F	TGGCCTTGTTCGATATACTGCGGA	813	44
			Og2-R	TCACGAGCTGAGCGAAACTGTTCA		
Og8	O8	*orf469*	Og8-F	CCAGAGGCATAATCAGAAATAACAG	448	43
			Og8-R	GCAGAGTTAGTCAACAAAAGGTCAG		
Og15	O15	*wzy*	Og15-F	TGGGCAATGGATTGGTATCT	608	45
			Og15-R	AGGGAAGAACACCGCTCCTAA		
Og18	O18	*wzy*	Og18ab-F	GTTCGGTGGTTGGATTACAGTTAG	551	43
			Og18ab-R	CTACTATCATCCTCACTGACCACG		
Og35	O35	*wzy*	Og35-F	TGCAGGTGCTTCAATTGGTT	303	45
			Og35-R	CCATCCAAATACGGAGCAATT		
Og78	O78	*wzx*	Og78-F	GGTATGGGTTTGGTGGTA	992	44
			Og78-R	AGAATCACAACTCTCGGCA		
Og88	O88	*wzy*	Og88-F	CTGCGCTTGGAGCATTCTAT	781	43
			Og88-R	GGCGCGAAACTTTCATATGC		
Og109	O109	*wzy*	Og109-F	GGATAATGGGGGTGGTTTTT	409	45
			Og109-R	GCTTCCCATCCTTGCAGATAT		

### E. coli serogrouping.

The same 100 virulent E. coli strains and the 87 nonvirulent E. coli strains used in antimicrobial susceptibility testing were also tested for nine serogroups associated with APEC: O1, O2, O8, O15, O18, O35, O78, O88, and O109. The identification frequency of serogroups for both nonvirulent and virulent E. coli strains can be found in Table 3. In total, only 23 out of 87 nonvirulent isolates and only 51 out of 100 virulent isolates belonged to one of the tested O-serogroups. No isolates from either group tested positive for the O1 serogroup. Virulent E. coli was associated more with serogroups O8 (31%) and O78 (11%) (*P < *0.001). Nonvirulent E. coli was more associated with serogroup O8 (9.2%), followed by serogroup O35 (8.05%) (*P < *0.004).

**TABLE 3:** Oxoid antimicrobial disk concentration, CLSI susceptibility range, and associated drug class for each antimicrobial used for the Kirby-Bauer disk diffusion assay (38)

Antimicrobial	Concn	Susceptibility E. coli ATCC 25922	Drug class
Chloramphenicol	30 μg	21–27 mm	Phenicol
Cephalothin	30 μg	15–21 mm	1st gen cephalosporin
Streptomycin	10 μg	12–20 mm	Aminoglycoside
Nalidixic acid	30 μg	22–28 mm	Quinolone
Erythromycin^*a*^	15 μg		Macrolide
Tetracycline	30 μg	18–25 mm	Tetracycline
Sulfamethoxazole/trimethoprim	25 μg	23–29 mm	Sulfa
Ampicillin	10 μg	15–22 mm	Penicillin
Cefoxitin	30 μg	23–29 mm	2nd gen cephalosporin
Ceftriaxone	30 μg	29–35 mm	3rd gen cephalosporin
Polymyxin B	300 μg	13–19 mm	Polymyxin

a Erythromycin does not have an effect on Gram-negative bacteria and was used as a negative control for E. coli isolates.

## DISCUSSION

### E. coli prevalence and virulence.

There was a very high prevalence of the five tested VAGs in E. coli isolated from NAE farms. The E. coli isolates collected from healthy broilers and environmental samples in NAE facilities in this study had a prevalence of VAGs similar to that of APEC isolates collected from colibacillosis-afflicted broilers in previous clinical studies (1, 17–20). For instance, in a study conducted by Kim et al., 79 APEC isolates collected from broilers with colibacillosis had a prevalence of VAGs at 79.7, 89.9, 93.7, 78.5, and 91.1% for genes *iroN*, *ompT*, *hylF*, *iss*, and *iutA*, respectively (20). With VAG prevalence similar to that of APEC as described by Kim et al., the current study indicated a possibility of high prevalence of APEC within NAE environment, thus exposing broilers to a greater risk of colibacillosis infection.

While the overall prevalence of APEC-like virulent isolates was high, growing season influenced the prevalence. Notably, the prevalence of samples with the five VAGs was highest in spring (80.6%), with a significant decrease into warmer months (13.0%; Fig. 2). Fluctuations in environmental temperature, humidity, and housing conditions greatly influence APEC prevalence. The steep decrease of virulent E. coli in warmer months (April to July) might be explained by decreased environmental humidity and increased tunnel ventilation, which is kept at a maximum to cool broiler house. These conditions reduce environmental moisture, litter water activity, and subsequent bacterial proliferation (21, 22). Dry conditions could limit APEC prevalence; however, more research is needed on the house conditions, local climate, and litter conditions throughout multiple seasons to be conclusive. Also, APEC prevalence could vary greatly in different geographical regions (11, 19, 23, 24).

It has been established that APEC can play a significant role in mortality in broilers at an early age (25). In contrast to our results, Varga et al. found no significant correlation between age group and VAGs in APEC isolates collected from colibacillosis-afflicted broilers (26). However, Pedroso et al. concluded that as chicken age increased, microbial diversity within chicken gastrointestinal tracts also increased (27). Conversely, multiple studies conclude that VAG diversity in broiler GIT declines as birds age, with young birds possessing a more diverse microbiota, possibly due to colonization from the surrounding environment (28–30). This fluctuation may reduce pathogenic E. coli within the poultry GIT over time, resulting in a change in VAG prevalence as flock age increases, as seen in Fig. 3. However, a combination of host, microbial, and environmental changes contribute to pathogenic APEC prevalence (31) within the current study, and more research in NAE on pathogenic E. coli prevalence and influencing factors such as flock age is needed.

The highest prevalence of VAGs, especially *ompT* and *iutA*, is associated with cloacal swabs and feces samples. In agreement with other studies, the current study suggests that the broiler GIT may serve as a reservoir for pathogenic APEC (28) and introduce pathogenic E. coli to the surrounding environment (32). Pathogenic E. coli could then be retained in the litter, and as broilers ingest, forage, and are in constant contact with the litter, litter is therefore an essential environmental factor that can contribute to disease (33). Litter has been shown to influence gut microbiota, with wet litter showing bacterial taxa similar to that of fecal samples (2). Reused litter may harbor pathogenic bacteria (34) and may increase the prevalence of VAGs in E. coli through horizontal transfer of virulent plasmids such as pAPEC-O2-R pAPEC-O2-ColV (35). Prevalence of *ompT* and *iutA* was 84.8% and 88.5%, respectively, in E. coli-positive litter samples, suggesting a high retainment of VAGs by E. coli in the surrounding environment.

### E. coli antimicrobial susceptibility.

There was greater frequency and degree of antimicrobial resistance seen in the virulent E. coli group than in the nonvirulent E. coli group. However, compared to that of APEC isolates from other studies, antimicrobial resistance in the current study was slightly lower (18, 20). For example, Mohamed et al. reported that 97.4% and 92.3% of APEC isolates were resistant to tetracycline and sulfamethoxazole/trimethoprim (18), respectively. However, only 62% and 29% of the virulent field E. coli isolates collected in this study were resistant to tetracycline and sulfamethoxazole/trimethoprim. This decrease in antimicrobial resistance in virulent E. coli is notable, suggesting a reduction in antimicrobial resistance in virulent E. coli isolates present in the NAE broiler environment compared to that in APEC isolates collected from conventional facilities (36). On average, virulent E. coli strains were resistant to seven antimicrobials and nonvirulent E. coli strains were resistant to only three. Multidrug resistance in APEC is associated with the transfer of antibiotic resistance genes (7). Phenotypic antimicrobial resistance observed in the highly virulent E. coli strains suggests that the NAE field E. coli isolates carry antimicrobial resistance genes and may contribute to colibacillosis infection in NAE broilers.

### E. coli serogroups.

Serogroup identification was successful in only 51% of virulent E. coli isolates and 26% of nonvirulent E. coli isolates. Of these, serogroup O8 was most prevalent in virulent isolates at 31%, followed by O78 at 11%. Our results are not consistent with APEC studies, as predominant serogroups were O1, O2, and O78 (1, 19, 37), whereas minimal O1 and O2 prevalence was found in the current study. The O78 was also the predominant identified serogroup in APEC isolates, with an occurrence of 17% or greater (1, 19, 20, 38). The prevalence of serogroup O8 is atypical of other recent APEC studies, in which no more than 2% of APEC was identified to be serogroup O8 (1, 19, 20, 39). More research is warranted in NAE and on the relationship between APEC and serogroup, as virulent E. coli was most associated with serogroup O8. If serogroup O8 continues to be the predominant serogroup in other NAE facilities as seen in this study, the O8 serogroup could elude producers to potential pathogenic E. coli prevalence within the NAE environment. Serogroup O35 was highly associated with nonvirulent E. coli and may not be related to pathogenic E. coli as once thought. In APEC studies, serogroup O35 was recovered in 1.5% or less of APEC isolates (1, 20, 38) and may not be a reliable serogroup to distinguish pathogenic E. coli from other environmental E. coli isolates.

### Conclusion.

In conclusion, there was a high prevalence of virulent E. coli within NAE farms. Virulent E. coli was notably higher in spring than in summer, suggesting that environmental factors influence VAG prevalence. Antimicrobial susceptibility testing revealed greater antimicrobial resistance in virulent E. coli than in nonvirulent E. coli, which may limit treatment options in colibacillosis outbreaks. Serogroups O78 and O8 were identified as the predominant serogroups in the virulent E. coli isolates. While O78 is consistent with other studies, serogroup O8 may be an important serogroup to monitor in NAE broilers. Considering the high virulence of E. coli, NAE producers should adopt measures to control colibacillosis outbreaks. These could potentially include vaccination, prebiotics and probiotics, enhanced water and feed hygiene, and housing management strategies.

## MATERIALS AND METHODS

### Experimental design.

Four commercial poultry farms from the same integrator under the NAE policy were selected in Mississippi. At each farm, two houses of mixed-sex broilers were chosen for sample collection. Sample collection was performed for two flock grow-outs, 61 days in length: one flock during the spring/winter months (February to April 2019) and one flock during the summer months (April to July 2019). Sample collection occurred on day 28 and day 56, consisting of litter, feces, cloacal swabs, and tracheal swabs.

All samplings in this trial were in compliance with the Guide for the Care and Use of Agriculture Animals in Research and Teaching (Federation of Animal Science Societies, 2010) and the Mississippi State University Institutional Animal Care and Use Committee (IACUC, Animal Welfare Assurance no. 17-224).

### Sample collection.

Eight litter samples, approximately 20 g each, were collected aseptically with a gloved hand from four quadrants of two poultry houses per farm on each sampling day. Litter was collected at no more than approximately 2.5 cm in depth in the house and placed into a 200 mL Whirl-Pak bag (Nasco Sampling/Whirl-Pak, Madison, WI, USA). Each sample was pooled from random spots within a quadrant and gloves were changed between samples. Similar sample collection was conducted for fecal samples collecting approximately 15 g per sample using sterile tongue depressors (SKU:25-705, Puritan, Guilford, Maine). Feces samples were collected from undisturbed droppings on the litter floor.

Using a similar sampling plan, eight cloacal swabs per farm were collected on each sampling day. A random, apparently healthy bird that was bright, alert, and active was selected in each quadrant and was swabbed using a sterile cotton swab (SKU: 25-806, Puritan, Guilford, ME). Briefly, the swab was placed into the cloaca of the bird, gently rotated clockwise around the inside the cloaca approximately three times, and immediately placed in a sterile culture tube (catalog no. 149569C Fisherbrand, Fisher Scientific, Pittsburgh, PA) containing 5 mL of buffered peptone water (BPW; Difco, Sparks, MD).

Eight tracheal swabs were collected from the same birds. While the bird was carefully restrained, the mouth of the bird was opened and the dry, sterile cotton swab was carefully inserted into the trachea via the opening of the larynx. The swab was inserted past the larynx gently, rotated clockwise three times, and then removed. This process was performed as gently and as quickly as possible to reduce stress to the bird. The tracheal swab was then immediately placed in a sterile culture tube (catalog no. 149569C Fisherbrand, Fisher Scientific, Pittsburgh, PA) containing 5 mL of BPW.

### Isolation and identification of Escherichia coli.

From collected litter, 10 g of litter was weighed aseptically and placed into a new 200 mL Whirl-Pak plastic bag with 90 mL of BPW, and the bag was stomached for 60 s. For fecal samples, 5 g of feces and 15 mL of BPW were used. Culture tubes containing cloacal and tracheal swabs in 5mL of BPW were vortexed for 30 s. Using a sterile inoculation loop (Fisherbrand, Fisher Scientific, Pittsburgh, PA), a loop full of suspension was streaked on duplicate MacConkey agar plates (Difco, Sparks, MD).

All MacConkey plates were incubated aerobically at 37°C for 24 h. Only colonies that appeared convex, smooth, pink, slightly mucoid, and sticky were selected (40). If present, two single colonies from each positive plate were transferred aseptically into culture tubes containing 3 mL of brain heart infusion (BHI; Difco, Sparks, MD). The culture tubes were incubated aerobically at 37°C for 24 h. Culture tubes were vortexed for 5 s. Then, 1.5 mL of the colony culture was placed into CryoELITE cryogenic vials (Wheaton Scientific Products, Millville, NJ) containing 400 μL of 80% glycerol and stored at −80°C and 150 μL was transferred to a 0.2 mL Pryme PCR microtube (MidSci, St. Louis, MO).

### DNA isolation of suspected E. coli isolates.

Microtubes were centrifuged for 3 min at 3,884 relative centrifugal force (rcf) in a VWR mini centrifuge. The supernatant was carefully discarded, and the pellet was not disturbed. Then, 150 μL of nuclease-free water (Fisher Bioreagents, Fair Lawn, NJ) was added back to the microtube containing the pellet. Microtubes were vortexed and heated at 98°C for 5 min. Microtubes were centrifuged again for 3 min. The supernatant containing DNA was then transferred to a new microtube. The prepared DNA templates were stored at 4°C for future use.

**Confirmation of** **E. coli** **isolates using qPCR.** We first confirmed isolates as E. coli by detecting the presence of *ybbW* gene, which is present in all E. coli strains, using forward primer 5′-TGATTGGCAAAATCTGGCCG-3′ and reverse primer 5′-GAAATCGCCCAAATCGCCAT-3′ (Eurofins Genomics, Louisville, KY) (41). The reaction mixture contained 5 μL PowerUp SYBR green (Applied Biosystems, Thermo Fisher Scientific), 0.25 μL of 10 μM forward primer, 0.25 μL of 10 μM reverse primer, and 3.5 μL of nuclease-free water for a total of 9 μL of template per reaction and 1 μL DNA template. Real-time PCR was run using a QuantStudio 3 (Applied Biosystems, Foster City, CA, US) using a 0.1 mL MicroAmp Fast 96-well reaction plate (Applied Biosystems, Life Technologies Corporation, Warrington, UK) and MicroAmp clear adhesive film (Applied Biosystems, Life Technologies Corporation, Warrington, UK). Real-time PCR parameters consisted of an initial denaturation at 95°C for 20 s, PCR stage of 40 cycles of 95°C for 1 s, 60°C for 20 s, with a melting curve analysis performed in a range of 60°C to 95°C at 0.5°C per 5 s increments. Confirmation of the *ybbW* was noted with a melt curve plot of an amplification wave seen at approximately 80.9°C in QuantStudio Design and Analysis Software v1.5.1.

Confirmed E. coli isolates were further screened for the presence of five VAGs, *iutA*, *iroN*, *hlyf*, *iss*, and *ompT*, as shown in Table 4 (1). The PCR mixture remained the same as listed above, except for the forward and reverse primers of each respective VAG. Real-time PCR parameters consisted of an initial denaturation at 95°C for 20 s, PCR stage of 40 cycles of 95°C for 1 s, 60°C for 20 s, with a melting curve analysis performed in a range of 60°C to 95°C at 0.5°C per 5 s increments.

**TABLE 4:** Number of virulent and nonvirulent E. coli isolates resistant to antimicrobials tested in this study using Kirby-Bauer disk diffusion assay; resistance was determined by susceptibility range established by Clinical & Laboratory Standards Institute (CLSI)^*a*^

Isolate virulence	*n*	No. of isolates resistant to:										
		AMP 10 (μg)	CEF 30 (μg)	CEFTRI 30 (μg)	CEPH30 (μg)	CHLOR 30 (μg)	ERYTH 15 (μg)	NA 30 (μg)	PB 300 μg	STREP 10 (μg)	SXT 25 (μg)	TET 30 (μg)
Nonvirulent	87	15	30	36	25	10	86	19	42	22	7	16
Virulent	100	30	23	48	32	13	100	23	45	39	29	62
												
Chi-square *P* value		0.042	0.082	0.364	0.629	0.915	0.282	0.850	0.558	0.046	0.001	<0.001

a AMP, ampicillin; CEF, cefoxitin; CEFTRI, ceftriaxone; CEPH, cephalothin; CHLOR, chloramphenicol; ERYTH, erythromycin; NA, nalidixic acid; PB, polymyxin B; STREP, streptomycin; SXT, sulfamethoxazole/trimethoprim; TET, tetracycline.

### Serogrouping of E. coli isolates using PCR.

Of the virulent E. coli isolates, those positive for all five VAGs, 100 were randomly selected, and all 87 nonvirulent E. coli (that contained none of the tested VAGs) were used for serogrouping and antimicrobial susceptibility testing. Isolates were tested for nine common serogroups historically associated with APEC: O1, O2, O8, O15, O18, O35, O78, O88, and O109. Serogroup reference strains used for gene confirmation and for PCR positive controls were acquired from the E. coli Reference Center, Department of Food Science, College of Agricultural Sciences, Pennsylvania State University. Serogroups and associated primers are listed in Table 5. The reaction mixture consisted of 0.25 μL of 10 μM forward primer, 0.25 μL of 10 μM reverse primer, 5 μL of PowerUp SYBR green, and 3.5 μL of nuclease-free water for a total of 9 μL per reaction and 1 μL DNA template. Real-time PCR was run for serogroups O8, O15, O18, O35, and O109 with parameters of an initial denaturation at 95°C for 20 s, followed by a PCR stage of 40 cycles of 95°C for 1 s, 60°C for 20 s, with a melting curve analysis performed in a range of 60°C to 95°C at 0.5°C per 5 s increments. Due to large gene size, traditional PCR was used to identify serogroups O1, O2, O78, and O88 in a Mastercycler (Model 5435 Mastercycler epgradient S, Eppendorf, Hamburg, Germany). The PCR mixture contained 0.25 μL of 10 μM forward primer and 0.25 μL of 10 μM reverse primer, 5 μL of GoTaq Green master mix 2× (Promega, Madison, WI), and 3.5 μL of nuclease-free water and 1 μL DNA template. PCR parameters consisted of an initial denaturation step of 95°C for 5 min, 40 cycles of denaturation at 95°C for 15 s, annealing at 57°C for 30 s, and extension at 72°C for 80 s, with a final extension at 72°C for 5 min. All PCR products were analyzed by agarose gel electrophoresis using a 1.5% agarose gel stained with SYBR Safe DNA gel stain (Invitrogen, Carlsbad, CA) in 1× Tris-acetate-EDTA (TAE) buffer. The results were visualized under UV light using the Kodak Gel Logic 200 Imaging System (Eastman Kodak Co., Rochester, NY).

**TABLE 5:** Number of virulent (isolates with all 5 virulence genes) and nonvirulent (isolates without any of the tested virulence genes) E. coli isolates belonging to each tested O-serogroup^*a*^

Isolate virulence	*n*	No. of isolates belonging to:									Total isolates identified
		O1	O2	O8	O15	O18	O35	O78	O88	O109	
Nonvirulent	87	0	1	8	0	3	7	0	1	3	23
Virulent	100	0	0	31	2	3	0	11	5	0	51
											
Chi-square *P* value			0.282	0.001	0.185	0.862	0.004	0.001	0.136	0.061	

a A total of 100 virulent and 87 nonvirulent isolates were tested against serogroups O8, O15, O18, O35, and O109 using real-time PCR and O1, O2, O78, and O88 using traditional PCR.

### Antimicrobial susceptibility testing of E. coli isolates.

The Kirby-Bauer disk diffusion assay (42) was used to test the antimicrobial susceptibility of the same 100 virulent E. coli isolates and all 87 of the nonvirulent E. coli isolates. Antimicrobial disks and associated susceptibility ranges for E. coli ATCC 25922 are listed in Table 1. E. coli isolates were inoculated into sterile culture tubes containing 3 mL BHI and incubated aerobically for 24 h at 37°C. Culture tubes were vortexed, 200 μL of the solution was transferred to 1.5 mL of sterile phosphate-buffered saline (PBS), and turbidity was adjusted against the 0.5 McFarland standard. A sterile cotton swab was dipped into the solution, and a Mueller-Hinton (Difco, Sparks, MD) plate was swabbed by sweeping the entire surface of the plate three times, rotating the plate 60° each time. A total of nine plates for each isolate were swabbed and allowed to dry for 5 min. Oxoid antimicrobial disks (Oxoid Ltd., Hants, UK) were spaced equally using sterile forceps. A total of 11 different antimicrobial disks were placed onto the three plates, and each isolate was replicated three times. Once all disks were placed, the Mueller-Hinton plates were incubated at 35°C for 18 h. The zone of inhibition (ZI) of each disk was measured to the nearest millimeter, and if no zone was present, the result was recorded as 0. Zones of inhibition were compared against the Clinical and Laboratory Standards Institute (CLSI) standards for antimicrobial susceptibility ranges (Table 1) (38).

### Statistical analysis.

For each virulence-associated gene, a proportion was calculated by dividing the number of samples that were positive for the gene by the total number of samples collected. Prevalence of virulent genes of E. coli was analyzed by the generalized linear model using GLIMMIX procedure of SAS 9.4 (Cary, NC) with season, day of age, and sample type serving as fixed effects. Frequency of serogroups for 100 randomly selected virulent E. coli isolates and 87 nonvirulent E. coli isolates was analyzed using an SAS procedure Chi-square test. Antimicrobial resistance of the same 100 randomly selected virulent E. coli isolates and the 87 nonvirulent E. coli isolates was analyzed by the general linear model (GLM) procedure, with ZI in millimeters as the dependent variable. The ZI was then determined as susceptible or resistant compared to the ATCC 25922 E. coli susceptibility range as seen in Table 1. Frequency of antimicrobial resistance for the virulent E. coli and nonvirulent E. coli groups was analyzed with Chi-square test in SAS 9.4. All analyses were performed by the SAS 9.4 software, with a significance level of 0.05.

## References

[1] Johnson TJ, Wannemuehler Y, Doetkott C, Johnson SJ, Rosenberger SC, Nolan LK. 2008. Identification of minimal predictors of avian pathogenic *Escherichia coli* virulence used for rapid diagnostic tool. J Clin Microbiol 46:3987–3996. doi:10.1128/JCM.00816-08. 18842938 PMC2593276

[2] Oakley BB, Lillehoj HS, Kogut MH, Kim WK, Maurer JJ, Pedroso A, Lee MD, Collett SR, Johnson TJ, Cox NA. 2014. The chicken gastrointestinal microbiome. FEMS Microbiol Lett 360:100–112. doi:10.1111/1574-6968.12608. 25263745

[3] Johnson JR, Russo TA. 2002. Extraintestinal pathogenic *Escherichia coli*: “the other bad *E. coli*”. J Lab Clin Med 139:155–162. doi:10.1067/mlc.2002.121550. 11944026

[4] Vandekerchove D, De Herdt P, Laevens H, Pasmans F. 2004. Colibacillosis in caged layer hens: characteristics of the disease and the etiological agent. Avian Pathol 33:117–125. doi:10.1080/03079450310001642149. 15276977

[5] Dziva F, Stevens MP. 2008. Colibacillosis in poultry: unravelling the molecular basis of virulence of avian pathogenic *Escherichia coli* in their natural hosts. Avian Pathol 37:355–366. doi:10.1080/03079450802216652. 18622850

[6] Nolan LK, Barnes HJ, Vaillancourt JP, Abdul-Aziz T, Logue CM. 2013. Colibacillosis, p 751–805. *In* Swayne DE, Glisson JR, McDougald LR, Nolan LK, Suarez DL, Nair V (ed), Diseases of Poultry, 13th ed. Iowa State Press, Iowa.

[7] Skyberg JA, Johnson TJ, Johnson JR, Clabots C, Logue CM, Nolan LK. 2006. Acquisition of avian pathogenic *Escherichia coli* plasmids by a commensal *E. coli* isolate enhances its abilities to kill chick embryos, grow in human urine, and colonize the murine kidney. Infect Immun 74:6287–6292. doi:10.1128/IAI.00363-06. 16954398 PMC1695531

[8] Mokady D, Gophna U, Ron EZ. 2005. Virulence factors of septicemic *Escherichia coli* strains. Int J Med Microbiol 295:455–462. doi:10.1016/j.ijmm.2005.07.007. 16238019

[9] Cloud SS, Rosenberger JK, Fries PA, Wilson RA, Odor EM. 1985. *In vitro* and *in vivo* characterization of avian *Escherichia coli*. I. Serotypes, metabolic activity, and antibiotic sensitivity. Avian Dis 29:1084–1093. doi:10.2307/1590463. 3914271

[10] Ewers C, Janssen T, Kiessling S, Philipp HC, Wieler LH. 2004. Molecular epidemiology of avian pathogenic *Escherichia coli* (APEC) isolated from colisepticemia in poultry. Vet Microbiol 104:91–101. doi:10.1016/j.vetmic.2004.09.008. 15530743

[11] Younis G, Awad A, Mohamed N. 2017. Phenotypic and genotypic characterization of antimicrobial susceptibility of avian pathogenic *Escherichia coli* isolated from broiler chickens. Vet World 10:1167–1172. doi:10.14202/vetworld.2017.1167-1172. 29184361 PMC5682260

[12] Ewers C, Janssen T, Kiessling S, Philipp H-C, Wieler LH. 2005. Rapid detection of virulence-associated genes in avian pathogenic *Escherichia coli* by multiplex polymerase chain reaction. Avian Dis 49:269–273. doi:10.1637/7293-102604R. 16094833

[13] Dibner JJ, Richards JD. 2005. Antibiotic growth promoters in agriculture: history and mode of action. Poult Sci 84:634–643. doi:10.1093/ps/84.4.634. 15844822

[14] Ritter GD, Acuff GR, Bergeron G, Bourassa MW, Chapman BJ, Dickson JS, Opengart K, Salois MJ, Singer RS, Storrs C. 2019. Antimicrobial-resistant bacterial infections from foods of animal origin: understanding and effectively communicating to consumers. Ann N Y Acad Sci 1441:40–49. doi:10.1111/nyas.14091. 30924543 PMC6850152

[15] Newman L. 2018. New strategies in coccidiosis control to meet customer demands. International Poultry Prod 26:15–17.

[16] Salois M. 2017. The cost of broiler welfare standard. 2017 Chicken Marketing Summit. Elanco Animal Health, Greenfield, IN.

[17] Schouler C, Schaeffer B, Bree A, Mora A, Dahbi G, Biet F, Oswald E, Mainil J, Blanco J, Moulin-Schouler M. 2012. Diagnostic strategy for identifying avian pathogenic *Escherichia coli* based on four patterns of virulence genes. J Clinical Microbiol 50:1673–1678. doi:10.1128/JCM.05057-11. 22378905 PMC3347144

[18] Mohamed L, Ge Z, Yuehua L, Yubin G, Rachid K, Mustapha O, Junwei W, Karine O. 2018. Virulence traits of avian pathogenic (APEC) and fecal (AFEC) *E. coli* from broiler chickens in Algeria. Trop Anim Health Prod 50:547–553. doi:10.1007/s11250-017-1467-5. 29164427

[19] Azam M, Mohsin M, Johnson TJ, Smith EA, Johnson A, Umair M, Saleemi MK, Rahman S. 2020. Genomic landscape of multi-drug resistant avian pathogenic *Escherichia coli* recovered from broilers. Vet Micro 247:108766. doi:10.1016/j.vetmic.2020.108766. 32768218

[20] Kim YB, Yoon MY, Ha JS, Seo KW, Noh EB, Son SH, Lee YJ. 2020. Molecular characterization of avian pathogenic *Escherichia coli* from broiler chickens with colibacillosis. Poult Sci 99:1088–1095. doi:10.1016/j.psj.2019.10.047. 32029145 PMC7587703

[21] Dumas MD, Polson SW, Ritter D, Ravel J, Gelb J, Morgan R, Wommack KE. 2011. Impacts of poultry house environment on poultry litter bacterial community composition. PLoS One 6:e24785. doi:10.1371/journal.pone.0024785. 21949751 PMC3174962

[22] Dunlop MW, McAuley J, Blackall PJ, Stuetz RM. 2016. Water activity of poultry litter: relationship to moisture content during grow-out. J Environ Manage 172:201–206. doi:10.1016/j.jenvman.2016.02.036. 26946169

[23] Solà-Ginés M, Cameron-Veas K, Badiola I, Dolz R, Majó N, Dahbi G, Viso S, Mora A, Blanco J, Piedra-Carrasco N, González-López JJ, Migura-Garcia L. 2015. Diversity of multi-drug resistant avian pathogenic *Escherichia coli* (APEC) causing outbreaks of colibacillosis in broilers during 2012 in Spain. PLoS One 10:e0143191. doi:10.1371/journal.pone.0143191. 26600205 PMC4657910

[24] Li Y, Chen L, Wu X, Huo S. 2015. Molecular characterization of multidrug-resistant avian pathogenic Escherichia coli isolated from septicemic broilers. Poult Sci 94:601–611. doi:10.3382/ps/pev008. 25667425

[25] Kemmet K, Williams NJ, Chaloner G, Humphrey S, Wigley P, Humphrey T. 2014. The contribution of systemic *Escherichia coli* infection to the early mortalities of commercial broiler chickens. Avian Pathol 43:37–42. doi:10.1080/03079457.2013.866213. 24328462

[26] Varga C, Brash ML, Slavic D, Boerlin P, Ouckama R, Weis A, Petrik M, Philippe C, Barham M, Guerin MT. 2018. Evaluating virulence-associated genes and antimicrobial resistance of avian pathogenic *Escherichia coli* isolates from broiler and broiler breeder chickens in Ontario, Canada. Avian Dis 62:291–299. doi:10.1637/11834-032818-Reg.1. 30339507

[27] Pedroso AA, Hurley-Bacon AL, Zedek AS, Kwan TW, Jordan AP, Avellaneda G, Hofacre CL, Oakley BB, Collet SR, Maurer JJ, Lee MD. 2013. Can probiotics improve the environmental microbiome and resistome of commercial poultry production? Int J Environ Res Public Health 10:4534–4559. doi:10.3390/ijerph10104534. 24071920 PMC3823317

[28] Kemmett K, Humphrey T, Rushton S, Close A, Wigley P, Williams NJ. 2013. A longitudinal study simultaneously exploring the carriage of APEC virulence associated genes and the molecular epidemiology of faecal and systemic *E. coli* in commercial broiler chickens. PLoS One 8:e67749. doi:10.1371/journal.pone.0067749. 23825682 PMC3692481

[29] Lu JR, Idris U, Harmon B, Hofacre C, Maurer JJ, Lee MD. 2003. Diversity and succession of the intestinal bacterial community of the maturing broiler chicken. Appl Environ Microbiol 69:6816–6824. doi:10.1128/AEM.69.11.6816-6824.2003. 14602645 PMC262306

[30] Crhanova M, Hradecka H, Faldynova M, Matulova M, Havlickova H, Sisak F, Rychlik I. 2011. Immune response of chicken gut to natural colonization by gut microflora and to salmonella enterica serovar enteritidis infection. Infect Immun 79:2755–2763. doi:10.1128/IAI.01375-10. 21555397 PMC3191970

[31] Casadevall A, Pirofski LA. 2001. Host-pathogen interactions: the attributes of virulence. J Infect Dis 184:337–344. doi:10.1086/322044. 11443560

[32] Clavijo V, Florez MJV. 2018. The gastrointestinal microbiome and its association with the control of pathogens in broiler chicken production: a review. Poult Sci 97:1006–1021. doi:10.3382/ps/pex359. 29253263 PMC5850219

[33] Kers JG, Velkers FC, Fischer EAJ, Hermes GDA, Stegeman JA, Smidt H. 2018. Host and environmental factors affecting the intestinal microbiota in chickens. Front Microbiol 9:235. doi:10.3389/fmicb.2018.00235. 29503637 PMC5820305

[34] Stanley VG, Gray C, Daley M, Krueger WF, Sefton AE. 2004. An alternative to antibiotic-based drugs in feed for enhancing performance of broilers grown on *Eimeria* spp.-infected litter. Poult Sci 83:39–44. doi:10.1093/ps/83.1.39. 14761082

[35] Johnson TJ, Siek KE, Johnson SJ, Nolan LK. 2006. DNA sequence of a ColV plasmid and prevalence of selected plasmid-encoded virulence genes among avian *Escherichia coli* strains. J Bacteriol 188:745–758. doi:10.1128/JB.188.2.745-758.2006. 16385064 PMC1347294

[36] Musa L, Proietti PC, Branciari R, Menchetti L, Bellucci S, Ranucci D, Marenzoni ML, Franciosini MP. 2020. Antimicrobial susceptibility of Escherichia coli and EBSL-producing Escherichia coli diffusion in conventional, organic, and antibiotic-free meat chickens are slaughter. Animals 10:1215. doi:10.3390/ani10071215. PMC7401526 32708915

[37] Rodriguez-Siek KE, Giddings CW, Doetkott C, Johnson TJ, Fakhr MK, Nolan LK. 2005. Comparison of *Escherichia coli* isolates implicated in human urinary tract infection and avian colibacillosis. Microbiology (Reading) 151:2097–2110. doi:10.1099/mic.0.27499-0. 15942016

[38] CLSI. 2016. Performance standards for antimicrobial susceptibility testing, 26th ed. Clinical and Laboratory Standards Institute, Wayne, PA.

[39] Paixao AC, Ferreira AC, Fontes M, Themudo P, Albuquerque T, Soares MC, Fevereiro M, Martins L, Correa de Sa MI. 2016. Detection of virulence-associated genes in pathogenic and commensal avian *Escherichia coli* isolates. Poult Sci 95:1646–1652. doi:10.3382/ps/pew087. 26976911

[40] Barnes H. J., Nolan L. K., Vaillancourt J. P., & Saif Y. M. (2008). Colibacillosis. *In* Saif YM (ed), Diseases of Poultry, p 691–732. Blackwell Publishing, Hoboken, New Jersey.

[41] Walker DI, McQuillan J, Taiwo M, Parks R, Stenton CA, Morgan H, Mowlem MC, Lees DN. 2017. A highly specific Escherichia coli qPCR and its comparison with existing methods for environmental waters. Water Res 126:101–110. doi:10.1016/j.watres.2017.08.032. 28930669

[42] Jan H. 2009. Kirby-Bauer disk diffusion susceptibility test protocol. American Society for Microbiology, Washington, DC. http://www.asmscience.org/content/education/protocol/protocol.3189.

[43] Li D, Liu B, Chen M, Guo D, Guo X, Liu F, Feng L, Wang L. 2010. A multiplex PCR method to detect 14 *Escherichia coli* serogroups associated with urinary tract infections. J Microbiol Methods 82:71–77. doi:10.1016/j.mimet.2010.04.008. 20434495

[44] Fratamico PM, Yan X, Liu Y, DebRoy C, Byrne B, Monaghan A, Fanning S, Bolton D. 2010. *Escherichia coli* serogroup O2 and O28ac O-antigen gene cluster sequences and detection of pathogenic *E. coli* O2 and O28ac by PCR. Can J Microbiol 56:308–316. doi:10.1139/w10-010. 20453897

[45] Iguchi A, Iyoda S, Seto K, Morita-Ishihara T, Scheutz F, Ohnishi M, Pathogenic E. coli Working Group in Japan. 2015. *Escherichia coli* O-Genotypic PCR: a comprehensive and practical platform for molecular O serogrouping. J Clin Microbiol 53:2427–2432. doi:10.1128/JCM.00321-15. 25926488 PMC4508431

